# Environmental injustice and childhood lead exposure in peri-urban (*ger*) areas of Darkhan and Erdenet, Mongolia

**DOI:** 10.1186/s12889-019-6486-x

**Published:** 2019-02-07

**Authors:** Erdenechimeg Erdenebayar, Keilah Dos Santos, Alexjandria Edwards, Nyam-Osor Dugersuren, Chimedsuren Ochir, Jerome Nriagu

**Affiliations:** 1 Department of Environmental Health, School of Public Health, Mongolian National University of Medical Sciences, Ulaanbaatar, 14210 Mongolia; 2 University of Michigan, Ann Arbor, MI USA; 3 School of Public Health, Mongolian National University of Medical Sciences, Ulaanbaatar, 14210 Mongolia; 4 Department of Environmental Health Sciences, School of Public Health, University of Michigan, Ann Arbor, MI48109-5406 USA

**Keywords:** Blood lead, Lead exposure, Environmental justice, Social inequity, Behavioral problems, Ger community, Erdenet, Darkhan

## Abstract

**Background:**

The *ger* (“tent city”) areas in Mongolia are a product of rapid urbanization and transitional economic development combine with lack of institutional, administrative and financial capacity of governments to cope with the pace. These areas have become traps for inequities in social and environmental services and the associated effects on human health. Disparities in childhood lead exposure in such communities are largely unexplored.

**Methods:**

We measured the concentrations of lead in blood of children, aged 4–7 years, in Erdenet (Orkhon Province) and Darkhan (Darkhan-Uul Province), the second and third largest cities in Mongolia. A survey instrument was used to gather information on influencing factors on lead exposure and the Strengths and Difficulties Questionnaire (SDQ) was used to assess a spectrum of behavioral problems among the children.

**Results:**

The mean blood lead level (BLL) of children in the two cities was found to be 3.8 ± 2.6 μg/dL (range: < 1.5–17.2 μg/dL) and 27.8% of the children had BLLs ≥5 μg/dL. Average BLL of children in Erdenet (a mining center) was significantly higher than that for children in Darkhan, and there was statistically significant difference between average BLL of children who live in *ger* district (4.2 ± 2.8 μg/dL) compared to those of children in housing units within the city (3.2 ± 2.4 μg/dL). In spite of the low values, BLLs was significantly associated with a number of effects on the spectrum of behavioral disorders, specifically with the scores for hyperactivity, conduct disorder and pro-social behavior.

**Conclusions:**

This study shows that childhood lead poisoning is common especially in *ger* communities of the urban areas of Mongolia. It contributes evidence showing that BLL low as 3.8 μg/dL can selectively activate some effects from a spectrum of likely behavioral disorders in children.

## Background

Although efforts have been made to reduce childhood lead poisoning in many countries, it remains a critical public health problem worldwide. Chronic exposure to low levels of lead has been associated particularly with neurodevelopmental toxicity in children which can be manifested as cognitive impairment, attention deficit or hyperactivity disorder and increased risk for antisocial and criminal behaviors [1–4]. These effects of early life exposure to lead may persist across the entire lifespan and result in decreased school performance, increased risk of drug abuse and incarceration, and proclivity to low income status (poverty) [4–7]. Lead poisoning affects not only the child’s health and well-being, but also the family’s economic well-being, job security, and stress level.

Epidemiologic data on childhood lead exposure in Mongolia is very limited. A 2005 screening of the blood of 120 children in Ulaanbaatar, the capital city of the country reported an average blood lead level (BLL) of 16.5 ± 9.5 μg/dL [8]. An earlier study of 153 children in 2014, also from Ulaanbaatar [9], found the BLL to be 6.0 ± 2.9 μg/dL and that 69.3% of the children had BLLs ≥5 μg/dL, the current reference level established by the United States Centers for Disease Control and Prevention [10]. These two studies with limited sample sizes are currently the only available (and reliable) information on environmental lead exposure and childhood lead poisoning in Mongolia. This study has expanded the bank of data on childhood lead exposure by presenting the first results ever on the BLL of children in Erdenet (Orkhon Province) and Darkhan (Darkhan-Uul Province), the second and third largest cities in Mongolia.

Like many developing countries, Mongolia has followed a pattern of rapid urban and economic development that has led to environmental, social and health inequalities in some communities [11–13]. A major contributor to disparities in lead exposure in the country is the spread of extractive industry, a highly exploitative enterprise in which the majority of the profits accrue to foreign mining companies while the burdens of environmental degradation and disease fall almost entirely upon local communities [14–16]. Another factor that contributes to inequitable exposure to lead pollution (environmental injustice) is the rapid urbanization and collateral growth of *ger* areas or shanty towns that extend for miles around the major cities [17–19]. Many people who live in *ger* districts are former herders, pulled into the city by the promise of a new life, or pushed off their land by mining operations, desertification and extreme winter weather [20–22]. About 45% of total households in the country now live in *ger* areas [23] which have become sprawling, unplanned settlements that lack basic services such as safe water supply, central heating, sanitation; paved roads and easy access to schools, markets, and health facilities [20, 24–26]. In the absence of central heating in the *ger* areas, most people rely on stoves both for cooking and heating. During winter, residents use large amounts of coal as well as other materials, including trash, to fuel the stove, resulting in a smoky cocktail of lead-containing pollutants, both indoor and outdoor [12, 27, 28]. By all indications, childhood lead poisoning should be a common feature in the *ger* settlements where environmental management is often non-specific, non-existent or poorly enforced [18, 29–31]. In addition, cultural practices, widespread cottage industries and poverty are likely to further heighten the likelihood of increased lead exposure in the shanty outskirts of the urban areas [31, 32].

In this cross-sectional study, we monitored the BLL of children in urban areas versus the *ger* districts of two major cities in Mongolia. We used a survey instrument to gather information on influencing factors on lead exposure and the Strengths and Difficulties Questionnaire (SDQ) [33] to assess a spectrum of behavioral problems among the children. The objective (BLL) and subjective (questionnaire) results were combined to explore the interconnected factors for inquitable exposure to lead and children’s behavioral problems in *ger* areas within the framework of environmental injustice. In the context of this paper, environmental justice refers to fair distribution of environmental benefits and burdens [34–36].

## Methods

Children for the study were recruited from the cities of Erdenet (Orkhon Province) and Darkhan (Darkhan-Uul Province). Erdenet has a population of 79,600 and is one of the youngest settlements in Mongolia. It was founded in 1974 in an area where large deposits of copper had been discovered in the 1950s. The city hosts the fourth largest copper mine in the world which produces 126,700 tons of copper and 1750 tons of molybdenum and accounts for about 13.5% of Mongolia’s gross domestic product [37]. Over the years, the mine has produced an enormous amount of tailings, estimated to be about 1 billion metric tons – a colossal environmental legacy in the city [30]. In the middle of the 1980s, more than 50% of the inhabitants were Russians working as engineers or miners. With the transition to market economy in the 1990s, most Russians left Erdenet. Today, the mine employs about 8000 people with much of the town being directly and indirectly affiliated with the mining sector. The city of Darkhan, on the other hand, was originally conceived to be a manufacturing site for Mongolia’s northern territory and was built, starting in 1961, with extensive economic assistance from the Soviet Union. The city remains a mostly industrial region and its population of 73,300 constitutes about 75% of the entire population of Darkhan-Uul Province. The *ger* district accounts for 26% of the population of the Darkhan soum or county [22]; the number of people that live in ger areas of the city of Erdenet is unknown (see Ref. [23]).

The population for this cross-sectional study consisted of a convenience sample of children, aged 4 to 7 years, in the two cities. Sample size of 300–350 was estimated using IBM SPSS Statistics assuming maximum margin of error of 5%, confidence level of 95%, the number of children 4–7 years old in the combined population of the two cities to be 4000 and a response rate of 60–70% (see https://www.surveysystem.com/sscalc.htm.

The study was conducted from May to June 2017. Community facilitators were used to identify elementary schools and kindergartens in selected areas in the urban and *ger* communities. The project staff then approached the Head of the selected school/kindergarten to get their permission to involve their schools in the study. The project next sought the help of teachers in the appropriate classes in distributing the study documents to children to take to their parents and getting them back. The package to parents included a letter inviting them to participate in the study, a consent form and a questionnaire, as well as a number to call if they had any questions. The signed consent form and completed questionnaire were returned to the school/kindergarten by the child. The project staff then coordinated with the teachers in the collection and analysis of blood samples from the children in a secluded area of the school. In addition to schools/kindergartens, a number of health clinics in urban and *ger* areas were contacted and agreed to the use of their facility in recruiting children for the study. Parents who visited the health clinic with children in appropriate age range were approached and asked to participate in the research study. Those willing to do so were asked to sign the consent form and the questionnaire was administered orally. The finger prick blood sample was collected and analyzed for lead by the project staff.

Blood lead levels were determined using the Lead Care II©, (**Magellan Diagnostics, Inc.,** Billerica, Massachusetts, USA). The Lead Care II© is a portable and inexpensive instrument which enables the lead in capillary blood to be analyzed conveniently in the field. It been certified for use by the CDC’s Lead and Multi-Elements Proficiency (LAMP) program and is an accepted method for monitoring blood lead to meet regulatory requirements. We closely followed the User’s Guide for Lead Care© [38] in the cleaning of the puncture point, in the collection blood and analysis of the sample. Approximately 50 μL of whole blood was collected from the children’s fingertip using a capillary tube provided in the test kit. During blood collection, the project staff (medical doctor or trained nurse) cleaned his/her hands with antiseptic soap and wore disposable gloves for personal protection and to prevent the external contaminants. According to the manufacturer, the quantification limits for Lead Care II© are 3.3 μg/dL to 65 μg/dL. Although lead was detectable below 3.3 μg/dL, the concentrations could not be quantified with much accuracy. The analyzer was calibrated each time a new lot of test kits was used. It should be noted that the US Food & Drug Administration (FDA) recently issued a warning on the use of Magellan’s diagnostics lead testing systems including LeadCare II [38] in lead poisoning prevention programs. The warning was based on data showing that test results on blood drawn from a vein may be lower than the actual level of lead in the blood. This warning, however, did not pertain to LeadCare Blood Lead Testing Systems when used with finger or heel prick (capillary) blood samples – as was the case in this study.

The structured questionnaire for this study was used to gather information on six domains covering location (city, soum and address);, child information (gender, age, height, weight, relationship to respondent); housing information (type, age, dustiness, distance from polluting source, heating and cooking system, type of stove, number of windows, plumbing, cleaning frequency); child health and risk behaviors (play area, mouth toys or suck thumb, school days missed, treatment with herbal remedies, wellness on a rating scale); parental risk behaviors (smoking, hobbies, pets, employment, use of glazed utensils); and socio-demographics of responding parent (gender, age range, marital status, educational attainment, height and weight). The effect of lead exposure on mental development of the children was assessed using the Strengths and Difficulties Questionnaire (SDQ). This short screening instrument of 25 items contains five different subscales measuring emotional symptoms, conduct problems, hyperactivity-inattention, peer relationship problems and prosocial behavior [33]. It is thus able to explore the psychological effects of lead poisoning in multiple dimensions. Each item was scored on a 3-point Likert scale with 1 = not true, 2 = somewhat true, 3 = certainly true. The score for each sub-scale was calculated by summing the score for constituent items with higher scores indicating greater problems, except the prosocial behavior, where a higher score indicated more positive behavior. A total difficulties score was obtained by summing the scores of the emotional symptoms, conduct problems, inattention-hyperactivity, and peer problems. Since SDQ is easy to use and authorized translations are available free of charge (see https://depts.washington.edu/dbpeds/Screening%20Tools/Strengths_and_Difficulties_Questionnaire.pdf), the instrument has been translated into more than 75 languages and extensively validated in clinical and community samples [39, 40].

Statistical analysis was performed with STATA12 (StataCorp, College station, TX,USA) and IBM SPSS 24 (Armonk, New York). The BLL and the scores for behavioral problems (SDQ scores) were the health outcome measures of interest in the model analyses. For numeric variables, the mean, median, standard deviation, and maximum and minimum concentrations were used to describe the distribution of the data. One hundred forty one (41.7% of total) had BLL < 3.3 μg/dL detection limit. For these cases, a value of 1.65 μg/dL (half the limit of quantification) was used in statistical calculations. An ANOVA was used for testing significant differences in BLLs between the different variables. The correlations between BLL (continuous variable) or SDQ scores (ranked variable) and the data for various categorical from from survey instrument were evaluated using Spearman’s rank correlation coefficient Spearman’s correlation coefficient. Bivariate correlations between the BLL and effector variables found to have *p*-values ≤0.05 were in included in multiple linear regression models used to further assess the relationships between BLL and the various risk factors. Statistical significance was considered for p-values less than 0.05.

The research plan, recruitment procedure and consent form were approved by the University of Michigan Institutional Review Boards (IRB#HUM00128035). The IRB Committee for the Mongolian National University of medical Sciences also approved the study (#2017/3–06). The parents or guardians of all children provided written informed consent after receiving detailed explanations of the study and recruitment letter.

## Results

Social-demographic characteristics of the children and parents and their effect on BLL are shown in Table 1. The mean BLL of children in the two cities was 3.85 ± 2.58 μg/dL (range: below detection limit (BDL) to 17.2 μg/dL) and 27.8% of the children had BLLs ≥5 μg/dL. It was found that BLLs of children in these two cities were lower than the recently reported BLLs of children, aged 7–14 years, in Ulaanbaatar (6.0 ± 2.9 μg/dL) [9]. A comparison of the BLL for children in Ulaanbaatar collected in 2005 (16.5 ± 9.5 μg/dL) versus 2014 (6.0 ± 2.9 μg/dL) suggests that the BLL of children have declined significantly since the introduction of unleaded gasoline in the country in 2008 [9, 41]. The difference in analytical methods used in the two studies – LeadCare II in 2014 versus atomic absorption spectrometry in 2005 – may also be a contributing factor to a discrepancy in reported results. We believe that the low BLLs of children in Darkhan and Erdenet are, in all likelihood, also related to the downward trend from the phase-out of leaded gasoline in Mongolia. Our result is similar to the most recent reference value of 3.48 μg/dL for children 1–5 years old in the United States [42]. They are also consistent with reported BLLs of children in other countries during the twenty-first Century. As examples, a review of BLLs in European countries found that the geometric mean (GM) values varied from 2 to 3 μg/dl [43]. Subsequent national surveys reported average BLLs of children to be 2.1 μg/dL in France [44] and 2.8 μg/dL in Greece [45]. A review by Li et al. [7]) reported that BLLs had dropped to < 5 μg/dL in 50% of the children in China.

**Table 1:** Blood lead levels of children and the social-demographic characteristics

Characteristics		N	Mean (SD, 95% CI)	Median (min. max)	*P*-value	% children with BLL ≥5 μg/dL
Total		338	3.85 (2.58, 3.6 4.16)	3.6 (<LOD,17.2)^a^		27.8 (94)
Gender	Boys	165	4.47 (2.99, 4.02 4.94)	4.1 (<LOD, 17.2)	0.001	35.7 (60)
	Girls	173	3.25 (1.94, 2.98 3.63)	3.3 (<LOD, 13.8)		20.0 (34)
Age(years)	4	93	3.73 (2.61, 3.22 4.29)	3.3 (<LOD, 17.2)	0.725	25.8 (24)
	5	84	3.96 (2.39, 3.49 4.68)	3.8 (<LOD, 16.4)		28.6 (24)
	6	72	4.03 (2.92, 3.34 4.71)	3.5 (<LOD,14.5)		27.8 (20)
	7	89	3.71 (2.43, 3.19 4.22)	3.3 (<LOD,13.8)		29.2 (26)
City	Darkhan	167	3.41 (2.15, 3.12 3.84)	3.3 (<LOD, 13.8)	0.032	22.2 (37)
	Erdenet	171	4.27 (2.88, 3.84 4.71)	3.8 (<LOD,17.2)		33.3 (57)
House type	Apartment	121	3.23 (2.36)	1.65 (<LOD,17.2)	0.0008	19.5 (23)
	*Ger* (tent)	217	4.18 (2.76)	3.9 (<LOD,16.4)		32.3 (71)
Parental education level	Primary	11	6.14 (2.44, 4.5 7.78)	5.7 (<LOD, 11.8)	< 0.0001	18.2 (2)
	Middle school	59	4.92 (3.72, 3.95 5.89)	4.0 (<LOD, 16.4)		36.2 (21)
	High school	106	3.97 (2.46, 3.50 4.45)	3.7 (<LOD, 13.8)		27.4 (29)
	College	30	3.5 (1.77, 2.83 4.16)	3.4 (<LOD, 7.0)		30.0 (9)
	Bachelor degree	129	3.24 (2.18, 2.86 3.62)	1.65 (<LOD,17.2)		20.0 (26)
Parents have a paying job	Yes	159	3.38 (2.12, 3.05 3.72)	3.3 (<LOD, 17.2)	0.0004	22.6 (36)
	No	174	4.40 (2.66, 3.95 4.85)	4.0 (16.4)		33.3 (58)

^a^: *LOD* is limit of detection

Average BLL was higher for male compared to female children (*p* = 0.001; Table 1), which is consistent with results of other researchers [46, 47]. There was no significant association between BLL and the age band in our study (Table 1). In contrast, results of many studies show that BLL increases with age, the explanation being that children tend to be more active and in contact with the environment, especially with the soil and dust as they grow older [48–50]. Average BLL of children in Erdenet was significantly higher than that for children in Darkhan (*p* = 0.032; Table 1), which can be attributed to the effect of lead pollution from the large mining operations in the former city. The genesis and living conditions in the ger districts of the two cities are similar, hence we decided not to treat them as separate entities in the data analysis.

Average BLL of children who live in *ger* district was 4.2 ± 2.8 μg/dL compared to 3.2 ± 2.4 μg/dL for children in housing units within the city; the difference in BLL for the two groups of children was statistically significant (*p* = 0.0008; Table 1).In addition, 32% of the children in *ger* areas had BLL ≥5 μg/dL compared to 20% for children in the city proper (Table 1). Other studies had also found that BLLs of children in Ulaanbaatar were significantly higher in peri-urban areas compared to the city proper [9, 51]. The BLL of children was correlated with parental educational level (*p* = 0.0001) and parents having a paying job (*p* = 0.0004) (Table 1). This should not be surprising considering that low income families were congregated in the *ger* areas outside the city and disproportionately rely on lower quality fuel (wood and coal) instead of electricity for heating and cooling [13, 25, 28].

We assessed the effects of various other influencing factors on BLL of the children (Table 2). Since the BLLs did not conform to normal distribution (skewness = 1.9; kurtosis = 5.2), they were log-transformed before they were used in subsequent regression models. The effect of child’s gender was statistically significant (p = 0.0001) with boys showing higher BLLs compare to girls but no correlation was found with children’s height, weight, or the number of children in the family (Table 2). Usage of herbal remedies (*p* = 0.004) and number of missed day from class in last 6 months due to illnesses were directly correlated with BLL (Table 2), implying that more people were relying on traditional medication in peri-urban areas. Association of usage of traditional medicine with increased BLL has been reported in several studies especially among the poor who cannot afford Western medicine [52, 53]. There was no correlation with other exploratory variables related to child health and child risk behaviors (Table 2). Also there was no correlation between BLL of children with parental risk behavior such as smoking (Table 2). parental education was negatively correlated with children’s BLL (*p* < 0.0001), similar to what has been reported in previous studies [54–56].

**Table 2:** Correlation of BLL with social risk factors of lead exposure

Correlation of child demographics with BLL									
Variables		Gender	Age			Weight	Height		#Children in household
BLL	Spearman’s Correlation	− 0.206	− 0.034			− 0.003	− 0.086		0.054
	p-value	0.0001	0.537			0.969	0.126		0.347
	N	334	335			335	334		309
Correlation of child health and risk behavior with BLL									
Variables		Playing outside within 100 m	Suck fingers	Mouth toys	Child has sickness	Use herbal remedies	Type of school attended	Missed school days	Child’s general health
BLL	Spearman’s Correlation	−0.045	−0.009	−0.098	− 0.022	0.169	0.124	0.210	−0.065
	p-value	0.406	0.877	0.074	0.685	0.004	0.032	0.026	0.243
	N	329	332	330	332	296	302	124	318
Correlation of parental risk behaviors with BLL									
Variables		Use of Glazed items		Lead related job or hobbies		Parental smoking		Any smoker in the home	
BLL	Spearman’s Correlation	−0.003		−0.012		−0.104		0.039	
	P-value	0.985		0.573		0.058		0.591	
	N	325		321		332		192	
Correlation of socio-demographics of parents with BLL									
Variables		Gender	Age	Marital status		Education level		BMI	
BLL	Spearman’s Correlation	−0.022	0.017	0.002		−0.236		0.057	
	P-value	0.694	0.771	0.971		< 0.0001		0.347	
	N	328	300	330		332		275	

Among the potential environmental risk factors, the type of housing (*p* = 0.001) and wood usage for heating (*p* = 0.009) and cooking (*p* = 0.008) were positively correlated, but centralized heating system (*p* = 0.052) and electricity usage for cooking (*p* = 0.026) were negatively correlated with BLL (Table 3). As to be expected, the year a *ger* was built was weakly associated with BLL (*p* = 0.077) where the age of a city dwelling was significantly (*p* = 0.013) correlated with BLL (Table 3).

**Table 3:** Correlation of BLL with various risk factors of lead exposure

Housing characteristics and BLL									
Variables		Type of house	< 500 m Distance from big industry		< 1 km from gasoline station	Distance from train station	Recent home renovation	Pet ownership	Remove shoes in the home
BLL	Spearman’s Correlation	0.198	−0.015		0.069	−0.059	0.052	− 0.086	0.118
	P-value	0.001	0.795		0.218	0.284	0.573	0.126	0.036
	N	335	315		322	329	226	315	319
Heating method and BLL									
		Coal	Wood	Agricultural waste	Natural gas	Heating oil	Electric wall board	Central heating system	
BLL	Spearman’s Correlation	0.008	0.144	0.054	−0.059	−0.052	− 0.004	− 0.107	
	P-value	0.885	0.009	0.329	0.285	0.341	0.946	0.052	
	N	335	331	331	331	331	331	331	
Fuel used for cooking and BLL									
		Coal		Wood	Agricultural waste		Natural gas		Electricity
BLL	Spearman’s Correlation	0.002		0.146	0.074		0.117		−0.130
	P-value	0.974		0.008	0.181		0.034		0.018
	N	330		331	331		331		331
Exposure risk factors for apartment residents and BLL									
Variables		Year structure wasbuilt	# Bed rooms	#window in the kitchen	Brass water meter	Frequency of home cleaned	Renovation house past 6 months	Water damage roof, walls	
BLL	Spearman’s Correlation	−0.244	− 0.098	− 0.146	0.055	− 0.064	0.052	0.199	
	P-value	0.013	0.297	0.123	0.555	0.492	0.573	0.033	
	N	105	113	111	115	115	116	113	
Exposure risk factors for *ger* area residents and BLL									
Variables		Year *ger* was built	Years in the home		Type of stove	Smoke meats	#window	Floor gets dusty	Frequency of home cleaning
BLL	Spearman’s Correlation	−0.126	−0.080		0.010	0.032	0.123	0.146	0.111
	P-value	0.078	0.231		0.887	0.651	0.107	0.037	0.115
	N	197	226		204	198	172	205	202

We used a linear regression model that included all variables from Tables 1-4 that showed significant associations with BLL in order to identify the most important mediating factors. The 12 variables together constituted a good predictor of BLL (df = 5; F = 11.8; r = 0.388; *p* < 0.001) even though they accounted for only about 15% of the variance in childhood BLL. The results of the regression model are shown in Table 4. The strongest predictors of BLL in the cities of Darkhan and Erdenet were the child’s gender (p < 0.001), education level of parents (*p* = 0.002), use of herbal remedies (*p* = 0.007), number school days missed because of sickness (p = 0.007) and whether the child lived in a ger tent or traditional building (*p* = 0.042). It would seem surprising that direct exposures from environmental sources are not featured prominently on the list of effector variables.

**Table 4:** Results of linear regression model showing the most important predictors of blood lead levels among the children in Darkhan and Erdenet

*Variable*	*Beta*	*t*	*p-value*
Child’s gender	−0.206	−4.047	0.000
Educational level (parent)	−0.181	−3.192	0.002
Missed school days	0.138	2.716	0.007
Use of herbal remedies	0.137	2.702	0.007
House type	0.117	2.046	0.042
Parents have a paying job	.052	0.922	0.357
Year housing structure was built	−.038	−0.753	0.452
Water damage (roof, wall)	.065	1.264	0.207
Remove shoes in the home	.020	0.369	0.713
Floor dustiness	.100	1.961	0.051
Heating with wood	.020	0.267	0.790
Cooking with wood	−.006	−0.093	0.926

## Discussion

Similar to observations in most countries, the removal of lead in gasoline sold in Mongolia has been significant in preventing lead poisoning or reducing the burden of lead in the blood of young children in the country. Even so, the reduction in lead exposure has not been equal for all children with those in the ger communities continuing to bear a disproportionate burden of exposure. This study endeavors to view the disparity in BLLs through the multidimensional lens of environmental justice which includes both the traditional exposure pathways and the quality-of-life factors (QOL) that may be predisposing children and their families in ger areas to lead poisoning. An environmental justice framework is discussed here as an approach that can be used to develop multi-level and multi-sectoral policies and partnerships needed to reduce and eliminate the burden of lead poisoning among the children in the country.

Previous studies in UB had concluded that contamination of local air and soils with lead from uncontrolled burning of coal for heating and cooking in households was primarily responsible for the difference in BLL of children in *ger* areas compared to the city proper [9, 51]. This conclusion remains conjectural as actual exposure to air pollution has not been assessed in previous studies. We did not find any significant association between the burning of coal for heating or cooling and BLL of children in Erdenet and Darkhan (Table 3). Enkhbat et al. [26]) likewise did not find any correlation between the BLL and burning of coal in their study of women who live in *ger* tents versus the city dwellers in UB. Apparently burning of coal does not appear to be the main effector of blood lead as people had previously thought. On the other hand, we found associations between BLL of children and indicators of socioeconomic status (parental level of education, parent have a paying job), low status on the energy ladder (heating with wood versus cooking with electricity), cultural values (use of herbal remedies), as well as disparities in type of house type (*ger* tent versus apartment) and where the residential structure is located (tent city versus city proper). In other words, the BLL is related to disparities in environmental and social-economic conditions of households in *ger* areas compared to the city proper as well as to higher disease burden in the *ger* communities (number of days that children missed school due to illness). We believe that these disparities and the mediating factors are a reflection of systemic injustices in the urbanization process in Mongolia [11, 11–20].

Lead poisoning contributes significantly to mental health problems among children in many poor communities (reviewed by [1, 57, 58]. In this study, we used parent-rated SDQ and linear regression models to explore the relationships between BLL and a spectrum of behavioral problems in the children (Table 5). We found that BLL was significantly associated with the score on hyperactivity (β = 0.433, t = 2.84, *p* = 0.007) and had weak effects conduct problems (β = 0.295; t = 1.99; *p* = 0.054) and pro-social behavior (β = 0.299; t = 1.99; *p* = 0.055) (Table 5). These effects were observed at the low average BLL (3.8 μg/dL) of the children that participated in this study. Our results reaffirm the notion that there is no safe blood lead concentration in children [10]. We did not find any association of BLL with emotional symptoms or peer problems contrary to the results of some previous studies [2, 4, 39, 58]. Age was only associated with pro-social behavior (*p* = 0.026) while the child’s gender was was only significantly associated with conduct problems (*p* = 0.008) (Table 5). The gender data are consistent with the observation that girls tend to display more emotional problems while boys have more externalizing issues such as conduct problems, hyperactivity/inattention, and peer problems [6]. Children in *ger* areas were at increased risk for emotional symptoms compared to those in the city proper (β = 0.137; t = 2.54; *p* = 0.012).. Similar effects on the spectrum of behavioral disorders have been reported in Belgian children in spite of the slight age differences (Belgian children were 7–8 years old) and much lower (1.4 μg/dL) blood lead values [39].

**Table 5:** Results of linear regression models showing the relationships between BLL and covariates with behavioral problems

Variables		Emotional symptoms	Conduct problems	Hyperactivity	Peer problems	Total difficulties	Pro-social behavior
Median SDQ score		4	3	5	5	16	7
*Ger* vs City proper	β	0.137	−0.041	0.035	−0.034	− 0.070	0.021
	t	2.54	−0.678	0.641	−0.603	−1.22	0.389
	*p*-value	0.012	0.498	0.522	0.546		0.698
Gender	β	0.0339	−0.145	0.002	−0.023	0.020	0.037
	t	0.547	−2.68	0.028	−0.410	0.362	0.678
	*p*-value	0.585	0.008	0.978	0.682	0.717	0.498
Age	β	0.065	−0.082	0.186	0.077	0.061	0.121
	t	0.400	−0.508	1.13	−0.455	0.373	1.24
	*p*-value	0.692	0.615	0.265	0.373	0.712	0.026
BLL	β	0.172	0.295	0.433	0.003	0.215	0.299
	t	1.12	1.99	2.84	0.023	1.21	1.99
	*p*-value	0.269	0.054	0.007	0.982	0.234	0.055

This is not the first study to associate BLL with behavioral problems among children (see, for instance, [3, 5, 10, 57]. It is, however, one of the first to treat childhood behavior as a multi-dimensional construct and to explore its relationships with BLL in a spectrum of behavioral disorders (specifically emotional response, conduct problems, hyperactivity/concentration, peer relations and pro-social behavior). We believe that discrepancies and controversies surrounding the results of previous studies on effects of lead on childhood behavior may stem partially from the treatment of this health outcome as being monotonous. Lead poisoning can induce a continuum of behavioral responses depending on the interplay of complex factors that drive the exposure-response relationships. An important observation from this study therefore is that lead exposure induces selective responses and does not affect the entire spectrum of possible outcomes in children equally and all at once. The multidimensional nature of the behavioral effects of lead poisoning therefore needs to be kept mind in any study that purports to “measure” the neurocognitive impairment from lead exposure.

*Ger* districts are a product of rapid urbanization and lack of institutional, administrative and financial capacity of governments in Mongolia to cope with the pace [13, 21, 29]. Their genesis can be traced to the reform package in the transition to market economy which called for privatization of state properties, elimination of state subsidies and expenditure, price liberalization, and dissolution of agricultural collectives and state farms [30]. These policies and programs brought social dislocations such as massive unemployment, soaring poverty, deteriorating social welfare, increasing corruption and deepening inequality. As more and more people in Mongolia were driven away from their familiar lands into the cities, the wealthy and the talented were readily assimilated while the underprivileged from rural areas found refuge in the outskirts of major cities including Erdenet and Darkhan. Additional impetus for migration to the urban filtering machine (separating the poor from the affluent) came in the form of increased desertification and extreme winter weather of 1999–2001 which wiped out a large proportion of the country’s livestock [30]. The impoverished herders of the *ger* areas became trapped and isolated both physically and socially from the rest of the city. Physically, they are not connected with the transportation, electricity and sewage systems of the main city [13, 20, 30]. Socially, they were treated as outcasts who were adding to the pressing urban problems and creating a tremendous burden on the economy, infrastructure and social safety net of the city proper [30]. The results of this study show that the economic growth, social development and spatial transformation of the cities have also come with costs which are borne disproportionately by children and environment in the contiguous peri-urban areas [59]. We conclude that the disparity in childhood lead exposure found in the *ger* communities falls within the rubric of environmental justice as it raises concerns for social justice and environmental protection in Erdenet, Darkhan and other urban areas of Mongolia.

A number of limitations in this study should be noted. First, it is difficult to prove causal relationship between lead exposure and health outcomes using a cross-sectional study design. Second, subjective evaluation of the children’s behavioral characteristics was provided by parents and recall bias cannot be completely ruled out. Third, there is potentially a selection bias in sampling for the study in schools, kindergartens and medical clinics which can limit the generalizability of the results to the entire cities. Fourth, the limit of detection for the LeadCare II instrument was only 3.3 μg/dL which made it impossible to examine the exposure-response relationships below this threshold. Out of the 338 children tested, 27.8% had BLL > 5 μg/dL implying that the study is not under-powered for the statistical analyses that were performed, however. To temper any potential bias from using only the values above the quantification limit, a median value of 1.65 μg/dL was assigned to any reading below 3.3 μg/dL. Finally, environmental samples were not collected that could be used to relate the BLL to sources of lead exposure and evaluate the exposure-response relationships.

## Conclusions

Childhood lead poisoning remains well-documented and pernicious yet a preventable global public health problem. This study shows that lead exposure remains common especially in peri-urban areas of Erdenet and Orkhon where nearly 30% of the children have blood lead levels above the current reference value of 5 μg/dL. Average blood lead level of children in our samples (3.8 μg/dL) are lower those found in the large metropolis of Ulaanbaatar (6.0 μg/dL). In spite of the relatively low levels of lead exposure, our study showed that the BLLs encountered in the two cities were having a significant effect on a spectrum of childhood behavior. As far as we know, this is the first study to examine the relationship between blood lead concentrations and behavioral problems in a large sample of Mongolian children. This study contributes further evidence showing that BLLs as low as 3.8 μg/dL can be a threat to children’s health. Despite the phase-out of leaded gasoline in Mongolia, many children are still showing up with relatively elevated BLLs that may be unsafe for them. There is clearly a need for a nationwide survey to assess the full extent of the problem of childhood lead exposure in the country, especially in marginalized peri-urban areas.

This study documents the fact that relative to the city proper, children in the *ger* districts are socio-economically disadvantaged and disproportionately exposed to lead (and other contaminants most likely). Such disparities in lead exposure at the individual and community levels likely have mediated the increased risk of behavioral problems among children in *ger* households. Comparatively speaking, these children can be considered to be victims of inherent environmental injustice in government policies and the urbanization processes of the two cities.

## Abbreviations

BDL: Below detection limit
BLL: Blood lead level
CDC: Centers for Disease Control (of the United States)
GM: Geometric mean
IRB: Institutional Review Board
LAMP: Lead and multi-element proficiency program
LOD: Limit of detection
SDQ: Strengths and difficulties questionnaire
UB: Ulaanbaatar
UNICEF: United Nations Children’s Fund
WASH: Water, Sanitation and Hygiene
WHO: World Health Organization

## References

[1] Grandjean P, Landrigan PJ (2014). Neurobehavioral effects of developmental toxicity. The Lancet Neurol.

[2] Lanphear BP, Hornung R, Khoury J, Yolton K, Baghurst P, Bellinger DC, Canfield RL, Dietrich KN, Bornschein R, Greene T, Rothenberg SJ, Needleman HL, Schnaas L, Wasserman G, Graziano J, Roberts R (2005). Low-level environmental lead exposure and children’s intellectual function: an international pooled analysis. Environ Health Perspect..

[3] Bellinger D, Leviton A, Allred E, Rabinowitz M (1994). Pre- and postnatal lead exposure and behavior problems in school-aged children. Environ Res..

[4] Liu JH, Liu XC, Wang W, McCauley L, Pinto-Martin J, Wang Y, Li L, Yan CH, Rogan WJ (2014). Blood Lead Levels and children’s Behavioral and Emotional Problems: A Cohort Study. JAMA Pediatr..

[5] Needleman HL, Schell A, Bellinger D, Leviton A, Allred EN (1990). The long-term effects of exposure to low doses of lead in childhood. An 11-year follow-up report. New Engl J Med..

[6] Holling H, Kurth B-M, Rothenberger A, Becker A, Schlack R (2008). Assessing psychopathological problems of children and adolescents from 3 to 17 years in a nationwide representative sample: results of the German health interview and examination survey for children and adolescents (KiGGS). Eur Child Adolesc Psychiat. 2008;[Suppl 1], 17:34–41. 10.1007/s00787-008-1004-1 19132302

[7] Li M-M, Cao J, Xu J, Cai S-Z, Shen X-M, Yan C-H (2014). The national trend of blood lead levels among Chinese children aged 0–18 years old, 1990–2012. Environ Internat..

[8] Burmaa B, Enkhtsetseg S, Baigal O, Oyunbileg D, Tsegmid S, Tuya S (2010). Environmental lead pollution in Ulaanbaatar City and its impact on children’s health. In: Burmaajav B and Enkhtsetseg S, ed. Environmental Health Research.

[9] Praamsma M, Ganbaatar N, Tsogtbaatar M, Halmambetova E (2016). Assessment of blood lead levels and associated risk factors among children in Ulaanbaatar. Mongolia. Central Asian J Med Sci..

[10] CDC. Low Level Lead Exposure Harms Children: A Renewed Call for Primary Prevention. Atlanta, GA: Centers for Disease Control and Prevention US Department of Health and Human Services; 2012. http://www.cdc.gov/nceh/lead/ACCLPP/Final_Document_030712.pdf. Accessed 7 January, 2018.

[11] Prasad A, Kano M, Dagg KA, Mori H, Senkoro HH, Ardakani MA, Elfeky S, Good S, Engelhardt K, Ross A, Armada F (2015). Prioritizing action on health inequities in cities: An evaluation of Urban Health Equity Assessment and Response Tool (Urban HEART) in 15 cities from Asia and Africa. Social Sci Med..

[12] Harpham T (2009). Urban health in developing countries: What do we know and where do we go?. Health & Place..

[13] Fan P, Chen J, John R (2016). Urbanization and environmental change during the economic transition on the Mongolian Plateau: Hoh hot and Ulaanbaatar. Environ Res..

[14] World Bank (2006). Mongolia: A Review of Environmental and Social Impacts in the Mining Sector May 2006.

[15] Morrice E, Colagiuri R (2013). Coal mining,social injustice and health: A universal conflict of power and priorities. Health & Place. 2013;19:74–79. 10.1016/j.healthplace.2012.10.006 23201912

[16] Jackson SL. Building a mineral nation: The Oyu Tolgoi Copper-gold mine and contested infrastructure development in Mongolia. PhD Thesis, Department of Geography, York University, Toronto, Ontario; 2014.

[17] UNDP (2011). Mongolia Human Development Report: From Vulnerability to Sustainability – Environmental and Human Health Development.

[18] Jadambaa A, Spickett J, Badrakh B, Norman RE (2015). The impact of the environment on health in Mongolia: a systematic review. Asia Pac J Public Health..

[19] Badarch D, Batsukh N, Batmunkh S, Badarch D, Zilinskas RA, Balint PJ (2003). The impacts of industrialization in Mongolia. Mongolia Today: Science, Culture, Environment and Development.

[20] Kamata T, Reichert J, Tsevegmid T, Kim Y, Sedgewick B (2010). Managing urban expansion in Mongolia: Best practices in scenario-based urban planning.

[21] Tsutsumida N, Saizen I, Matsuoka M, Ishii R (2015). Addressing urban expansion using feature-oriented spatial data in a peripheral area of Ulaanbaatar. Mongolia. Habitat Internat..

[22] Herro M. Darkhan Demographic Report. USAID/CHF Supporting Enterprises and Economic Development (SEED) Program, USAID Cooperative Agreement No. AID 492-A-00-Q2-Q0017–00, Darkhan, Mongolia; 2003.

[23] National Statistics Office of Mongolia. The 2015 Population and Housing By-census of Mongolia: Annual Report. Ulaanbaatar, Mongolia. 2016;2016 http://www.1212.mn/BookLibraryDownload.ashx?url=hun_am_oron_suutsnii_2015_toollogo_eng.pdf&ln=Mn. Accessed 20 November 2017.

[24] Sigel K, Altantuul D, Basandorj K (2012). Household needs and demand for improved water supply and sanitation in peri-urban ger areas: the case of Darkhan. Mongolia. Environ Earth Sci..

[25] World Bank (2011). Air Quality Analysis of Ulaanbaatar: Improving Air Quality to Reduce Health Impacts.

[26] Enkhbat U, Rule AM, Resnick C, Ochir C, Olkhanud P, Williams DL (2016). Exposure to PM2.5 and Blood Lead Level in Two Populations in Ulaanbaatar, Mongolia. Int. J. Environ. Res. Public Health..

[27] Nagniin S, Janchiv O, Palam E, Jalkhaa K (2011). Air pollution and health Ulaanbaatar city of Mongolia. Epidemiology..

[28] Nakao M, Yamauchi K, Ishihara Y, Omori H, Ichinnorov D, Solongo B (2017). Effects of air pollution and seasons on health-related quality of life of Mongolian adults living in Ulaanbaatar: cross-sectional studies. BMC Public Health..

[29] Uddin SMN, Li Z, Gaillard JC, Tedoff PF, Mang H-P, Lapegue J, Huba EM, Kummel O, Rheinstein E (2014). Exposure to WASH-borne hazards: A scoping study on peri-urban Ger areas in Ulaanbaatar. Mongolia. Habitat Internat..

[30] Myadar O (2009). Nomads in a fenced land: Land reform in post-socialist Mongolia. Asian-Pacific Law & Policy Journal..

[31] Nriagu JO, Blankson ML, Ocran K (1996). Childhood lead poisoningin Africa: a growing public health problem. Sci Total Environ..

[32] Nriagu JO, Jinabhai CC, Naidoo R, Coutsoudis A (1997). Lead poisoning of children in Africa, II. Kwazulu/Natal, South Africa. Sci. Total Environ..

[33] Goodman R (1997). The strengths and difficulties questionnaire: a research note. J Child Psychol Psychiatry.

[34] Vickery J, Hunter LM (2016). Native Americans: Where in Environmental Justice Research?. Soc Nat Resour..

[35] Johnson T, Lora-Wainwright A, Lu J (2018). The quest for environmental justice in China: citizen participation and the rural-urban network against Panguanying's waste incinerator. Sustain Sci..

[36] McDonald YJ, Jones NE (2018). Drinking Water Violations and Environmental Justice in the United States, 2011-2015. Am J Public Health..

[37] MRPAM. Mineral Resources and Petroleum Authority of Mongolia Annual Report. Ulaanbaatar-15171, Mongolia. 2017. https://www.mrpam.gov.mn/public/pages/66/MPRAMreport2016EN.pdf Accessed 21 Feb, 2018.

[38] Magellan Diagnostics, Inc. LeadCare II Blood Lead Analyzer User’s Guide. 101 Billerica Ave, N. Billerica, Massachusetts 01862–1271 USA. https://www.magellandx.com/leadcare-products/leadcare-ii/support/product-literature/.

[39] Sioen I, Hond ED, Nelen V, Van de Mieroop E, Croes K, Van Larebeke N, Nawrot TS, Schoeters G (2013). Prenatal exposure to environmental contaminants and behavioral problems at age 7–8 years. Environ Internat..

[40] Moriwaki A, Kamio Y (2014). Normative data and psychometric properties of the strengths and difficulties questionnaire among Japanese school-aged children. Child Adolesc Psychiat Mental Health.

[41] US EPA. Partnership for Clean Fuels and Vehicles: Evaluation of the Design and Implementation of the Lead Campaign. Report EPA-100-R-11-008, Office of Policy, United States Environmental Protection Agency, Washington, D.C.; 2011. https://www.epa.gov/sites/production/files/2015-09/documents/pcfv-eval-final-report.pdf. Accessed 6 Nov 2017

[42] Caldwell KL, Cheng P-Y, Jarrett JM, Makhmudov A, Vance K (2017). Laboratory measurement implications of decreasing childhood blood lead levels. Pediatrics..

[43] Jakubowski M. (2011) Low-level environmental lead exposure and intellectual impairment in children – the current concepts of risk assessment. Internat J Occupat Med Environ Health. 2011;24: 1–7. 10.2478/s13382-011-0009-z 21468897

[44] Etchevers A, Bretin P, Lecoffre C, Bidondo ML, Le Strat Y (2014). Blood lead levels and risk factors in young children in France, 2008–2009. Internat J Hygiene Environ Health..

[45] Kapitsinou A, Soldatou A, Tsitsika A, Kossiva L, Tsentidis Ch, Nisianakis P, Theocharis S, Garoufi A (2015). Risk factors for elevated blood lead levels among children aged 6–36 months living in Greece. Child Care Health Developm. 2015;41: 1199–1206 10.1111/cch.12254 25968465

[46] Counter SA, Leo H (2015). Buchanan, Fernando Ortega. Blood lead levels in Andean infants and Yong Children in Ecuador: International Comparison. J Toxicol Environ Health..

[47] Chen J, Li M, Lv Q, Chen G, Li Y (2015). Blood lead level and its relationship to essential elements in preschool children from Nanning, China. J Trace Elements Med Biol.

[48] Jiang Y-M, Shi H, Li J-Y, Shen C, Liu J-H, Yang H (2010). Environmental lead exposure among children in Chengdu, China: Blood Lead levels and Major Sources. Bull Environ Contam Toxicol..

[49] Wu Y, Yang X, Ge J, Zhang J (2011). Blood lead level ant its relationship to certain essential elements in the children aged 0 to 14 years from Beijing. China. Sci Total Environ..

[50] Li Y, Wu S, Xiang Y, Liang X (2014). An investigation of outpatient children's **b**lood lead level in Wuhan China. PLoS One..

[51] Dorogova VB, Burmaa B, Enkhtsetseg B (2008). Environmental lead pollution in Ulan-Bator and children’s health. Gigiena i sanitariia.

[52] Shen X, Rosen JF, Guo D, Wu S (1996). Childhood lead poisoning in China. Sci Total Environ..

[53] Courtney JG, Ash S, Kilpatrick N (2002). Childhood Lead Poisoning Associated with Tamarind Candy and Folk Remedies ---California, 1999–2000. MMWR Morb Mortal Weekly Report..

[54] Albalak R, Noonan G, Buchanan S, Flanders WD, Gotway-Crawford C, Kim D, Jones RL, Sulaiman R, Blumenthal W, Tan R, Curtis G, McGeehin MA (2003). Blood lead levels and risk factors for lead poisoning among children in Jakarta. Indonesia. Sce Total Environ..

[55] Mohammad H. Ranbar, Maureen Samms-Vaughan, Aisha S. Dickerson et al (2015) Factors associated with blood lead concentration of children in Jamaica J Environ Sci Health A Tox Hazard Subst Environ Engr. 2015;50(6):529–536 10.1080/10934529.2015.994932 PMC4659644 25837555

[56] Ruzanna G, Petrosyan V, Melkomian DM, Khachadourian V, McCartor A, Crape B (2016). Risk factors for children’s blood lead level in metal mining and smelting communities in Armenia: a cross-sectional study. BMC Public Health..

[57] Rauh VA, Margolis A (2016). Research Review: Environmental exposures, neurodevelopment and child mental health – new paradigms for the study of brain and behavioral effects. J Child Psychol Psychiatry..

[58] Jedrychowski W, Perera FP, Jankowski J, Mrozek-Budzyn D (2009). Very low level prenatal exposure to lead and mental development of children in infancy and early childhood: Krakow prospective cohort study. Neuroepidemiology..

[59] UNICEF (2017). Mining-related in-migration and the impact on children in Mongolia - Research Findings and recommendations. United Nations Children’s Fund (UNICEF).

